# Use of menstrual cups among school girls: longitudinal observations nested in a randomised controlled feasibility study in rural western Kenya

**DOI:** 10.1186/s12978-018-0582-8

**Published:** 2018-08-17

**Authors:** Anna Maria van Eijk, Kayla F. Laserson, Elizabeth Nyothach, Kelvin Oruko, Jackton Omoto, Linda Mason, Kelly Alexander, Clifford Oduor, Aisha Mohammed, Alie Eleveld, Isaac Ngere, David Obor, John Vulule, Penelope A. Phillips-Howard

**Affiliations:** 1 Department of Clinical Sciences, Liverpool School of Tropical Medicine (LSTM), Pembroke Place, Liverpool, L3 5AQ UK; 2 Centre for Global Health Research, Kenya Medical Research Institute (KEMRI), Kisumu, Kenya; 3 Division of Global Health Protection, Center for Global Health, Centers for Disease Control and Prevention (CDC), Atlanta, USA; 4 Department of Obstetrics and Gynaecology, Siaya District Hospital, Ministry of Health, Siaya, Kenya; 5 Division of Reproductive Health, Ministry of Health, Nairobi, Kenya; 6 Safe Water and AIDS Project, Kisumu, Kenya; 7 Ministry of Health, Siaya County, Kenya

**Keywords:** Schoolgirls, Menstrual cup, Menstrual hygiene management, Silicone, Mooncup, Adolescents, Sexual and reproductive health, Africa, Kenya

## Abstract

**Background:**

A menstrual cup can be a good solution for menstrual hygiene management in economically challenged settings. As part of a pilot study we assessed uptake and maintenance of cup use among young school girls in Kenya.

**Methods:**

A total of 192 girls between 14 to 16 years were enrolled in 10 schools in Nyanza Province, Western Kenya; these schools were assigned menstrual cups as part of the cluster-randomized pilot study. Girls were provided with menstrual cups in addition to training and guidance on use, puberty education, and instructions for menstrual hygiene. During repeated individual visits with nurses, girls reported use of the menstrual cup and nurses recorded colour change of the cup.

**Results:**

Girls were able to keep their cups in good condition, with only 12 cups (6.3%) lost (dropped in toilet, lost or destroyed). Verbally reported cup use increased from 84% in the first 3 months (*n* = 143) to 96% after 9 months (*n* = 74). Colour change of the cup, as ‘uptake’ indicator of use, was detected in 70.8% of 192 participants, with a median time of 5 months (range 1–14 months). Uptake differed by school and was significantly higher among girls who experienced menarche within the past year (adjusted risk ratio 1.29, 95% CI 1.04–1.60), and was faster among girls enrolled in the second study year (hazard ratio 3.93, 95% CI 2.09–7.38). The kappa score comparing self-report and cup colour observation was 0.044 (*p* = 0.028), indicating that agreement was only slightly higher than by random chance.

**Conclusions:**

Objective evidence through cup colour change suggests school girls in rural Africa can use menstrual cups, with uptake improving with peer group education and over time.

**Trial registration:**

ISRCTN17486946. Retrospectively registered 09 December 2014.

**Electronic supplementary material:**

The online version of this article (10.1186/s12978-018-0582-8) contains supplementary material, which is available to authorized users.

## Plain English summary

Girls in developing countries with few means to take care of themselves can find it hard to attend school during menstruation. The menstrual cup can be a good solution for dealing with menstruation in areas where alternatives such as sanitary pads are expensive or scarce. However, people have been concerned if the menstrual cup is acceptable for young girls in these settings. As part of a larger study looking into the use of menstrual items in rural Africa, school girls were given menstrual cups. They got puberty education and training on how to use them and keep them in good condition. They visited a nurse regularly; during these visits they talked about cup use and the cup was inspected for damage and colour change, a potentially more objective measure of cup use. Generally, girls were able to maintain their cups in good condition, with only 12 cups lost. More girls used the cup over time, showing that it takes a while to get used to the cup. There was low agreement between what girls said they did (use the cup) and if the cup had a colour change (looked like it was being used). Peer education helped girls get used to the cups, and cups of girls enrolled in the second study year showed colour change much faster.

## Background

Menstrual hygiene management (MHM) for school-aged girls in low and middle income countries can be challenging when they have limited funds for, and access to, high quality hygiene products and poor sanitary conditions to deal with menstruation [1]. Use of inferior products, such as unhygienic cloths or prolonged use of a material before changing, can cause skin irritation, restriction in movement, and concerns about leaking and odour in school [2, 3]. Inadequate MHM has been associated with an increased risk of urogenital infections [4, 5]. Inability to deal with the blood flow reportedly can lead to absenteeism at school or the workplace, although data are difficult to interpret [2, 6–9]. Qualitative studies note girls’ stigma if menstrual blood leaks at school, resulting in an inability to focus on lessons and withdrawal from school activities [2, 3, 10]; these studies also suggest that lack of options for MHM leads to school absenteeism and drop-out [2, 10, 11]. Both qualitative and quantitative studies indicate that girls value modern menstrual products [2, 3, 12–14], such as branded sanitary pads; in some settings transactional sex is used to obtain these products from boys and men [2, 5, 12]. Improved MHM may thus be considered an essential component among a variety of interventions to strengthen girls’ sexual and reproductive health, can reduce their sexual exposures, and increase their chance of reaching their potential in school [1].

An alternative to sanitary pads is the menstrual cup, which has received attention in relatively small-scale studies in high income [15–18], and low and middle income countries, including among schoolgirls [5, 19–22]. Made of high grade medical grade silicone, rubber, latex or elastomer, these bell-shaped receptacles collect menstrual flow when inserted into the vaginal canal, and can be emptied and reinserted with a need to boil the cup only at the end of a cycle [23, 24]. Cups have the advantage of reuse, and can potentially last up to 10 years. First introduced in the 1930’s, there are now ~ 100 brands available worldwide – marketed as an eco-friendly and cost-saving approach to menstrual care [23]. Cups have been shown to be safe with no incumbent infection risk among European [15, 16] and North American women [17]. Preliminary studies of acceptability in low and middle income countries suggest cups are a potential option for girls as well as women [19, 20, 25]. Studies to date have reported outcomes based on women and girls’ self-reported cup use, but have not physically evaluated actual cup use or their robustness for sustained use in impoverished settings where environmental hazards (extreme temperatures, household pests) may reduce shelf-life [7]. A systematic review of MHM products recommended more rigorous ascertainment of cup use before large scale trials are conducted to determine cost-effectiveness [26].

As part of a pilot study examining the feasibility and safety of menstrual cup use on outcomes such as school attrition, and sexual and reproductive infections among primary schoolgirls who have reached menarche in rural western Kenya, [5, 24] our previous qualitative study demonstrated girls’ reported cup use and acceptance [22]. In the current quantitative study, we utilise colour change of the cups as a more objective measure of uptake and use by girls, and compare this against girls’ reported use, and examine factors that appear to influence the time taken for girls’ to start using the cup [5].

## Methods

### Setting

The study was carried out in Gem District, Siaya County, nested within a wider health and demographic surveillance system (HDSS), which follows a population of ~ 230,000 individuals, described elsewhere [27]. Gem covers a rural area of 300 km^2^, with its southernmost point ~ 12 km from Lake Victoria. The population are mostly members of the Luo ethnic group, and are mainly subsistence farmers [28]. Siaya is an impoverished area [29]: while the wider (former Nyanza) Province has a literacy rate of 70%, compared to the national average of 62%, school surveys estimate four out of 10 child learners miss school daily in Siaya County [30]. The gender equity occurring in primary school falls during adolescence, with a quarter more boys than girls attending secondary school by 18 years of age [31, 32]. The public health burden is typical of rural African communities [27]; past mortality among adolescents and young adults has been largely ascribed to communicable diseases and injuries [33], and maternal mortality among females [34]. Gender-based violence is one of the highest in Kenya, with 22% of women reporting to have ever experienced sexual violence (compared to 14% for the whole country) [35].

### The menstrual solutions study

The research presented is part of a menstrual feasibility study, a single-site, three-arm, open cluster randomized controlled ‘proof of concept’ pilot study [5]. In brief, 30 of 62 primary schools in the area included in a water, sanitation and hygiene (WASH) survey, were selected based on minimum WASH criteria [36]. The 30 primary schools were randomized into three groups; menstrual cups, sanitary pads, or usual practice. Girls from these schools were eligible to participate if aged 14–16 years, if they lived in the study area, received parental consent, assented, experienced three or more menses, and had no reported disability precluding participation. The study examined the acceptability, use, and safety of menstrual products, and social and schooling experiences of girls followed over one academic year. This paper focuses exclusively on girls’ experience of the menstrual cup in the 10 schools randomly allocated to the cup group. Findings on main outcomes [5], focus group discussions [22], the laboratory confirmed safety of the cups, examining *Staphylococcus aureus* infection, toxic shock syndrome toxin-1, and cup contamination [24], and water, hygiene, and sanitation associated with MHM in the schools [36–38], have been published elsewhere.

### The menstrual cup

Eligible girls in the menstrual cup schools were provided with one Mooncup® size B for nulliparous women (Additional file 1: Figure S1). This brand was selected because it has been tested in the UK [15, 16] and internationally [16, 25], is produced to ISO 13485:2003 standards (a regulated quality management system requiring that the service provided by an organisation “consistently meet[s] customer and regulatory requirements applicable to medical devices and related services”), and is registered by the U.S. Food and Drug Agency of Medicines (FDA; Registration Number 3009117944). The white colour of the cup when new, changing to light brown after use, allowed for physical observation of use. According to the manufacturer, when inserted into the vaginal canal the cup collects ~ 30 ml of menstrual blood, lasting 4–8 h depending on flow before emptying is required [39]. While a variety of menstrual cups are available for sale through distributors in Kenya, none were available or marketed at the time in the rural study area.

### Safety precautions

Participants, families, communities, schools, and health facilities received written information in English and the local language about the Menstrual Solutions Study, the menstrual cup, symptoms of toxic shock syndrome, and contact information for the research team and local village community health worker. Guidance on toxic shock syndrome was provided in the information sheets, and contact details given should girls experience any symptoms. At each school, girls elected a favoured teacher to be the study focal point to strengthen communication between research nurses, schools, participants, and the research team. Research study nurses were each allocated a cluster of 3–4 study schools comprising at least one school from each intervention arm, and were responsible for monitoring the health and wellbeing of study participants within these schools. Soapy water for handwashing was made available for all participating schools. Other safety assessments over time are reported elsewhere [40].

### Participant training

All participating girls received a puberty and hygiene classroom lesson from the school-allocated study nurse. For girls in the cup arm, this included an anatomical and practical base for cup-specific training. Study nurses were first provided with menstrual cups for their own use, and had a training session with the study gynaecologist, and with the menstrual hygiene WASH expert. Nurses then gave classroom training on cup use and safety to girls in the 10 cup-allocated schools. Training included how to fold and insert the cup, how to remove and empty it, how to keep it clean and boil at the end of each cycle, and how to store it to prevent loss or damage. Nurses reinforced use and safety messages, and requested girls communicate directly with them if they had any problems. Written materials were provided in addition to classroom demonstrations. A rolling enrolment of participants took place across two study years, between August 15, 2012 and August 27, 2013 with menstrual cups provided shortly after enrolment. Nurse assessment visits of participants occurred between October 2012 and November 2013. Early feedback from informal discussions with participating girls and focal point teachers indicated girls were reluctant to start using their cup. As a consequence, peer-to-peer classroom training was conducted by Luo ‘champion’ secondary schoolgirls from a school in a contiguous area, who had received cups from a charity in the previous 6 years and demonstrated expertise with use, in all cup schools in October 2012.

### Monitoring cup use

Study nurse clinical assessments of each participant privately but within the school campus were planned for a minimum of once per term per girl, with nurses visiting their schools on average once per week. However, it was not possible to conduct these at predetermined intervals due to logistical issues, such as schools not wanting girls to be assessed on certain days as well as girls being absent or too busy with school lessons or activities on the days that nurses visited. Nurses had face-to-face discussions with individual girls about the use of their assigned menstrual product to ascertain use, hygiene requirements, and any problems encountered. Girls in the menstrual cup arm were asked specific questions on their cup use, if they dropped it, shared use with another person, and how they cleaned their cup. Girls were requested to bring in their cup (barring current use) for physical examination at each screening visit, whereby aspects of the cup were documented by the reporting nurse, including colour change, tail length, damage, detritus and smell (Additional file 1: Table S1).

### Outcome measures and data analysis

All survey data were captured through field netbook entries. After cleaning, data were imported, transformed and analysed using Stata (version 14). Data were merged with girls’ family socio-economic status, derived from multiple component analysis recorded in their household, with 5 quintiles of poorest to least poor, dichotomised into poorest (quintile 1–2), and less poor (3–5), and girls with missing data were included as a third category [5]. Evidence of cup use was defined by observed change in colour of the cup from clear/white to yellow/brown at nurse assessment. Additional physical observed evidence of use was a trimmed stem of the cup (see Additional file 1: Figure S1), which could demonstrate that girls had adapted the cup for their own size. Further evidence was girls’ self-reported use to a study nurse during the assessment. Total follow up time for each participant was calculated as the date of cup receipt to the date of the final nurse assessment visit, or the date of reported loss of cup or cup replacement. Characteristics of girls who verbally reported cup use or not were compared using generalized estimating equations with a logit link, and exchangeable correlation [41]. Characteristics of girls with cups showing colour change were compared with girls whose cups did not change over the study period using Pearson’s χ^2^ test and Fishers Exact test if small numbers, and generalized linear regression with a log link and binomial distribution [42]; covariates examined included age, socio-economic status, class, years since menarche at time of cup receipt, total follow up time and number of assessment visits. Models were adjusted for clustering by school. The Mann-Whitney test was used to compare the follow up time among girls with and without a cup colour change. Non-parametric survival analysis was conducted using Kaplan-Meier survival analysis to plot time to event (first visit with colour change of the menstrual cup or first verbal report of cup use); the log-rank test was used to compare the plots. Cox regression was additionally conducted to examine differences in time to colour change by the covariates mentioned above with school as cluster variable. To evaluate the relationship between verbal responses on cup use with the observed changes in the cup, a variable was created which showed the verbal response at the time of first report of cup colour change if this occurred, and the verbal response at the last nurse assessment visit if the colour change did not occur. We used the kappa score to compare self-report and cup colour observation; if there would be complete agreement this score would be 1, whereas for no agreement other than what would be expected by chance, the score would approximate 0. A *p*-value of < 0.05 was considered statistically significant.

### Ethical considerations

The Menstrual Solution Study was granted ethical approval from the Scientific and Ethical Review Boards of the Kenya Medical Research Institute and the Ethics Committee of the Liverpool School of Tropical Medicine, and was considered CDC “non-engaged” by the Center for Global Health of the U.S. Centers for Disease Control and Prevention. Parents provided written informed consent and girls gave their written informed assent; participants were informed they had the right to withdraw at any time.

## Results

### Characteristics of the study population

Among the 267 girls in the 10 schools allocated to the menstrual cup arm, 60 were excluded because they did not meet the eligibility criteria for the study, and 7 girls did not want to participate (after they received a cup) [5]. Among the 207 participating girls who received a cup, 192 presented their cups for viability checks at least once, and comprised the study population for this nested sub-study. The majority of girls were in class 7 at the time of enrolment (56.3%, Table 1), with a mean age of 14.6 years. Twelve percent of girls were in households with the poorest two quintiles. Most girls (87.2%) had started their menstruation before reaching 15 years; the mean duration of menses was 3.7 days (standard deviation 1.7) and only 10% had menses longer than 5 days. About one in five girls said their menses was heavy, and the majority of girls (62.0%) reported cramps when menstruating. Before enrolment in the study, 88% reported they had (ever) used commercial sanitary pads.

**Table 1:** Characteristics of study participants, Menstrual Solutions Pilot study, Western Kenya, 2012–2013

Characteristic at enrolment	Number (%), *N* = 192
Class at time of enrolment	
5	14 (7.3)
6	47 (24.5)
7	108 (56.3)
8	23 (12.0)
Age at enrolment, years	
14	93 (48.4)
15	80 (41.7)
16	19 (9.9)
Mean age (sd), years	14.6 (0.7)
Socio-economic status household of girls^a^	
Poorest 2 SES quintiles	22 (11.5)
Higher 3 SES quintiles	138 (71.9)
No information	32 (16.7)
Age at menarche in years^b^	
Mean age at menarche (sd)	13.5 (1.0) *n* = 188
< 13	20 (10.6)
13	63 (33.5)
14	81 (43.1)
15	24 (12.8)
Mean days of bleeding (sd)	3.7, sd 1.4, range 2–14
Days of bleeding	
≤ 5	172 (89.6)
6–7	18 (9.4)
> 7	2 (1.0)
Amount of bleeding	
Heavy	40 (20.8)
Medium	96 (50.0)
Light	56 (29.2)
Cramps when menstruating	Any: 119 (62.0)
Mild cramps	59 (30.7)
Moderate cramps	30 (15.6)
Severe cramps	30 (15.6)
Material used for menstruation before enrolment	
Used some pads	169 (88.0)
Used cloths	21 (10.9)
Used other items	2 (1.0)

Abbreviations: *sd* standard deviation

^a^The socio-economic status of the household was calculated using a weighted average from multiple correspondence analyses (MCA), whereby indicators were generated from biennial household surveys in the Health and Demographic Surveillance System [5]

^b^Unknown for 4 girls

### Follow up of participants

Participants were followed up for a median of 10.9 months (IQR 6.1–12.5), with a median of four (range 1–12) cup assessment visits. Ten percent of participants were seen once only by the study nurse. Three quarters (74.5%) of girls had their first nurse visit within 3 months after the receipt of the cup. The median duration of physical cup observation follow-up was 7.4 months, ranging from 1 to 14 months, slightly shorter than the median participation in the study. Twenty-eight participants stopped observations before the end of the study and the reasons included pregnancy (14), marriage (1), migration (7), withdrawal (1), and ‘other’ (5).

### Self-reported menstrual cup use, problems with use and maintenance

**Fig. 1:**
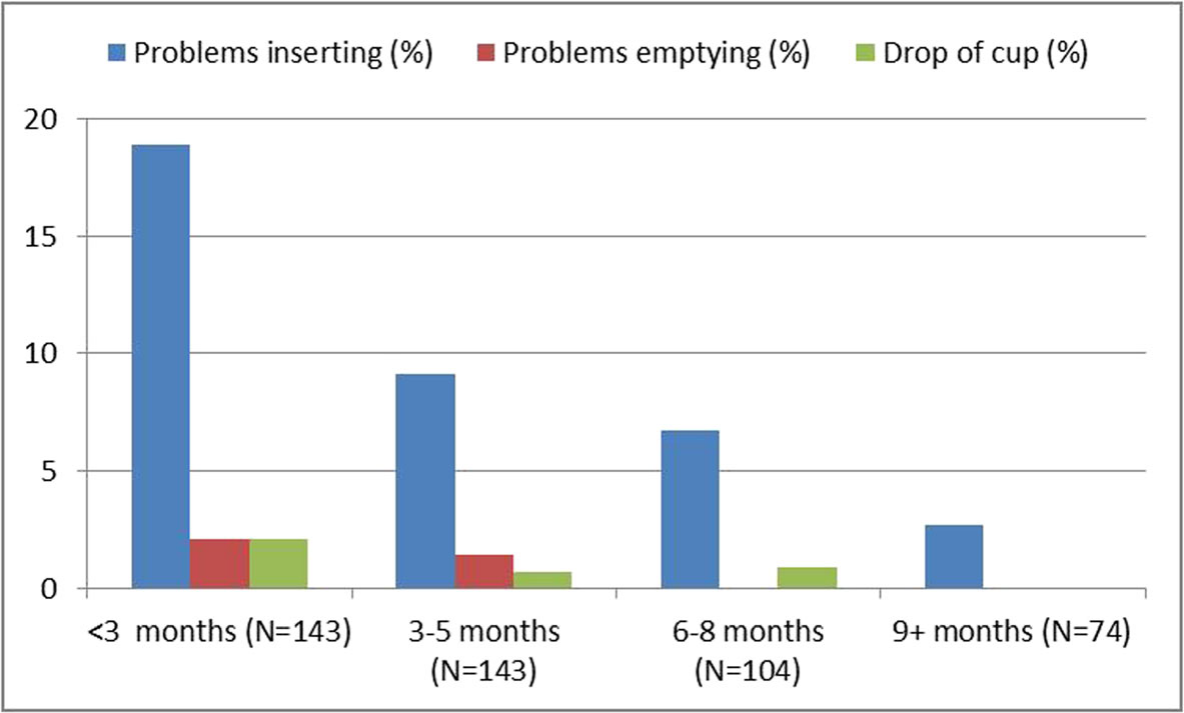
Menstrual cup problems over time, Western Kenya, 2012–2013

During the nurses assessment visits, 83.9% of 143 girls screened within 3 months stated they had used the cup, and this increased to 96.0% after 9 months (Additional file 1: Figure S2). Problems with cup use as reported to the nurses, such as with insertion or emptying, decreased from 21% among girls in first 3 months of provision, to 3% after 9 months (Fig. 1) whereas most girls reported good habits with regard to hygiene (Additional file 1: Figure S3). A list of complaints and issues reported to the nurses can be found in the Additional file 1: Table S2. None of the girls reported sharing the cup with other girls or women. When exploring factors associated with a verbal report of cup use among all study visits (Additional file 1: Table S3), girls were more likely to self-report cup use if they had started their menstruation in the previous year compared to girls who had menstruated for more than a year (RR 2.03, 1.04–3.94, *p* = 0.04, adjusted for school), and less at a visit in the first 3 months (0.38 0.19–0.79, *p* = 0.010 compared to a visit after 9 months). Self-report of cup use varied significantly by school (Additional file 1: Table S3).

### Observed cup viability

During the follow up visits to the study nurse, only minor defects were detected on cups which did not result in the need for cup replacement (Additional file 1: Figure S3). Two girls required new cups due to extensive damage (one eaten by rat and one burnt during too long boiling). Thirteen girls within this cohort followed required a new cup during the study because they had dropped the cup in the toilet or pit latrine (7), or somewhere else (3), or leaking occurred (3: replaced with a bigger cup).

### Cup colour change

A colour change of the cup was recorded after observation by nurses for 136 of 192 participants (70.8%); the median time to first colour change was 5 months (range 0.3–13.8 months, mean 5.6 months, standard deviation 3.3), with cups of 34 girls (25%) having colour change within 3 months, 44 (32.4%) within 4–6 months, 35 (25.7%) within 7–9 months and 23 (16.9%) after 9 months. Girls without a cup colour change were more likely to have left the study prematurely (26.8% vs. 9.6%, *p* = 0.002). The median total follow-up time for girls with a cup colour change was 8.9 months (range 1–13.8), whereas this was 4.1 (range 1.1–13.8) among the 56 girls without colour change of the cup (Mann-Whitney test *p* < 0.001). Girls without a cup colour change were more likely to become pregnant (6/56 vs. 3/136, *p* = 0.04 Fisher exact test), more likely to migrate (4/56 vs. 3/136, *p* = 0.22 Fisher exact test) and more likely to drop out (4/56 vs. 1/136, *p* = 0.05 Fisher exact test); there was no difference by enrolment year. A significantly shorter follow up time among girls without a cup colour change remained after excluding girls who left the study prematurely.

The uptake of cup use as assessed by colour change of the cup differed significantly by school (Additional file 1: Figure S4, chi-square test *p* < 0.001) and was significantly more common among girls who started menstruation recently at the time of enrolment (< 1 year, 84.3%) compared to girls who menstruated for a longer time (≥1 year, 66.0%, chi-square test *p* = 0.013). This remained significant in multivariate analysis when adjusted for number of follow up visits and duration of follow up (adjusted risk ratio 1.29, 95% CI 1.04–1.60, *p* = 0.023) (Additional file 1: Table S4 for full analysis). Time to cup colour change was significantly associated with year of enrolment with a faster colour change among girls who were enrolled in the second study year (hazard ratio 3.93, 2.09–7.38, *p* < 0.001, Table 2).

**Table 2:** Factors affecting time to colour change of the menstrual cup, as observed during visits to the study nurse, Western Kenya, 2012–2013

Variable	*n*	Univariate Hazard ratio, 95% CI^a^	*p*-value	Multivariate Hazard ratio, 95% CI^a^	*p*-value
Class at time of enrolment					
5 or 6	61	0.73, 0.57–0.94	0.013	NS	
7 or 8	131	Reference			
Age at enrolment (years)					
14	93	Reference		NS	
15	80	1.17, 0.88–1.56	0.268		
16	19	3.81, 1.59–9.14	0.003		
Socio-economic status household of girls					
Poorest 2 quintiles	22	1.12, 0.73–1.72	0.589	Not included	
Higher 3 SES quintiles	138	0.90, 0.70–1.15	0.410		
No information	32	Reference			
Material used for menstruation before enrolment					
Used some pads	169	Reference		Not included	
Cloths/other	23	0.92, 0.48–1.73	0.788		
Time since menarche at enrolment					
< 1 year	51	1.01, 0.63–1.62	0.958	Not included	
≥ 1 year	141	Reference			
Enrolment year					
2012	126	Reference		Reference	
2013	66	3.93, 2.09–7.38	< 0.001	3.93, 2.09–7.38	< 0.001
Enrolled before/after peer education^b^					
Before peer education	102	Reference		NS	
After peer education	90	2.77, 1.25–6.13	0.012		

*CI* confidence interval, *NS* not significant in multivariate model

Note: a hazard ratio > 1 indicates a shorter duration to cup colour change

^a^School included as cluster variable

^b^Peer-to-peer classroom training conducted by ‘champion’ secondary schoolgirls from a school in a contiguous area in October 2012

### Observed cup colour change and self-reported cup use

**Fig. 2:**
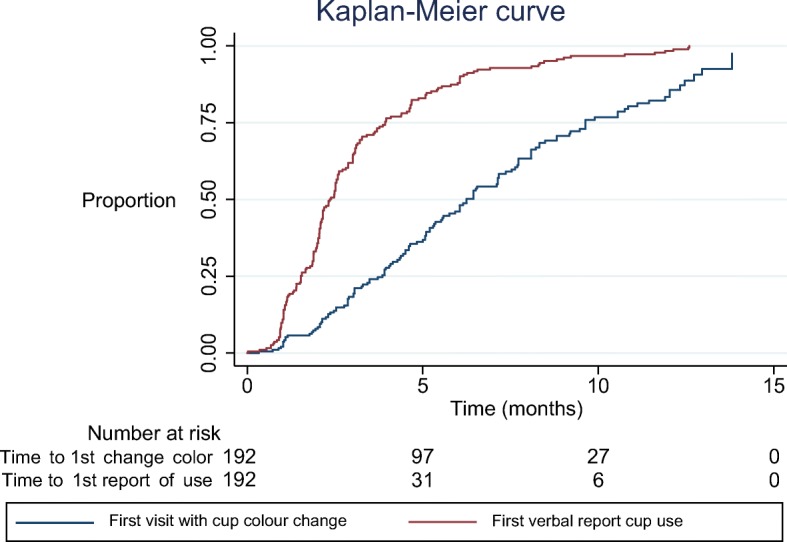
Kaplan Meier curve of time to first verbal confirmation of menstrual cup use and time to first detected colour change of the menstrual cup. Note: Log rank test: *p* < 0.001

There was a discrepancy between reported cup use and observed cup use from colour change of the cup or physical change such as the cup stem being cut for comfort, with reported use much higher than any of the other measures (Additional file 1: Figure S2 & S4). Self-reported use was at least once by 187 out of the 192 girls (97.4%) and increased faster than a colour change of the cup (70.8% in total, Fig. 2). Among the 136 girls with a cup colour change, 131 (96.3%) said they used the cup at the visit that the colour change was noted; however, 89.3% of girls whose cup did not changed colour (50/56) said they used it as well (*p* = 0.06). The girls with a self-report of cup use but without a cup colour change were not significantly different by age, school class or socio-economic profiles compared to girls with a cup with colour change, but were less likely to be in their first year of menstruation (Additional file 1: Table S5). The kappa score comparing self-report and cup colour observation was 0.044, which indicates that the agreement was only slightly higher than by random chance.

## Discussion

### Main findings

Among 192 young school-going girls in Kenya (mean age 14.6 years) provided with menstrual cups in addition to training and guidance on use, puberty education and instructions for MHM, colour change of the cup as an indicator of use was detected in 70.8%. Verbal reports of cup use did not correspond well with colour changes of the cup; verbally reported cup use increased from 84% in the first 3 months (*n* = 143) to 96% after 9 months (*n* = 74) whereas cup colour change was detected among 22% and 74% in the same time periods, respectively. Time to cup colour change was faster among girls enrolled in the second year of the study (hazard ratio 3.93, 95% CI 2.09–7.38) who might have had a greater amount of peer support. Uptake differed by school and was higher among girls who experienced menarche more recently (adjusted OR 1.29, 95% CI 1.04–1.60). Girls were able to keep their cups in good condition, with only 12 cups (6.3%) lost (dropped in toilet, lost or destroyed).

### Interpretation

Few studies have evaluated the potential use of an insertable menstrual product among schoolgirls in an economically impoverished setting. The current study presents unique objective evidence depicting a conservative estimate of actual use, based on biologically-plausible physical changes to the menstrual cup acquired over time, and compares this against girls’ self-reported cup use. These data show that cup uptake was gradual, with initial resistance, as reported in other studies [43–45], requiring intervention through mentorship. Our research team sought advice from Luo girls in a secondary school in Siaya County, who were experienced menstrual cup users (courtesy of long-term charity provision). Their assistance as champions for peer-to-peer training of our study girls helped to initiate cup use; this was only significant as a variable in univariate but not in the multivariate Cox regression model and not in any other model. The importance of the peer group may be indirectly postulated by the difference seen in uptake (verbal and by cup colour change) by school, and illustrate the strong social component, including peers and teachers. The faster uptake among girls enrolled later in the study may be a consequence of the presence of peers familiar with cup use; the importance of peers was also observed in study among school girls in Nepal and Uganda [21, 45]. Presence of study nurses, strong research communications, and goodwill among the schools and community to examine this unmet need for schoolgirls may have strengthened resolve to participate. During the follow-up in this study, we detected no evidence of health or hygiene risks associated with menstrual cup use, similar to previous studies [15–17, 40].

Verbal self-reported responses showed higher and faster uptake compared with a more objective measure using colour change as an approximation of use. While menstrual flow may have an effect on time to cup colour change, we note manufacturers indicate cups’ colour will change from white to light brown following use; however, such a colour change may not occur with coloured cups. Cup colour change was used here as a binary variable; there was no evidence of a gradual darkening of the cup over increased use, thus we were unable to analyse as a ‘dose-response’. Our results suggest programs and research screening on use should be cautious when relying on self-reported use only to assess the impact of cups on recipients’ lives. This is especially important for trials using menstrual cups where, in absence of a more objective measure, the impact is under-estimated because ‘cup users’ are over-estimated. For example, in a per-protocol analysis of the data of the feasibility pilot [5], the greatest impact of cups on sexually transmitted infections and bacterial vaginosis was after girls had been provided the cups for 9 months or longer. Among girls with discoloured cups the measured outcomes of dropout and sexually transmitted infections (combined outcome) were halved (relative risk [RR] 0.48; 95% confidence limits 0.25–0.91) if they had the cup for 9 months, compared with controls; while for girls with no cup colour change having the cup for at least 9 months there was no difference from controls (RR 1.42; 0.48–4.18) [5]. This significant outcome difference adds credibility to cup-colour change as a predictor of cup use. In addition, from our and other studies introducing menstrual cups, it is clear that cup provision alone (without supportive training) is inadequate to ‘pull’ girls and women over the threshold to actual use, and that peer and other ongoing support is critical, especially in the early months following provision [21, 44].

### Strengths and limitations

We used colour change of the cup as an indicator of use but there are no scientific directions on the gradation of cup discoloration with use over time from the manufacturer, or guidelines. While some biases may occur with heavy or light menstrual flow, we consider this measure as more objective than self-reported use or observation of the trimming of the tail of the cup, supported by differences in ‘impact’ effects depending on observed cup colour change. Discoloration has been described for menstrual cups and diaphragms, without decrease of effectiveness or life span, and has been ascribed to effects of local vaginal conditions [46, 47]. It is possible that vaginal infections may affect colour change; we asked girls for symptoms but there was no agreement between reported symptoms and infections identified. No girls complained to nurses that they could not use the menstrual cup due to any ongoing symptoms. Colour change was reported for some girls very soon after receipt of the cup, so a noticeable colour change may occur early. Colour change of the cup during use can be reverted when it is put for a couple of hours in sunlight [47]; however, the school girls were instructed to carefully put their cup away in a pouch in between menses after boiling, to avoid theft or loss, and their verbal reports indicated they hid the cup to prevent others using it. In addition, girls and women are often embarrassed to leave menstrual materials in the open, and usually they are cleaned, dried and stored out of view, despite guidelines for e.g. cloths to dry in sunlight [8]. We note girls were not informed we would use cup colour change as an indicator of use. We cannot exclude that girls treated the cup in other ways that may have reverted the colour, but we are not aware of such methods. It is possible some girls may have stopped using the cup after colour change; however, other studies have shown once use is initiated, girls and women report continued use [17, 21]**.** The cups were distributed to girls as they were enrolled, reducing follow up time to a short duration for a few girls who were recruited late. The nurses’ assessments of some girls (due to their absence, or school events) were less regular. This may have led to an under-estimation of the duration required until the cup colour changed, and may have contributed marginally to a conservative estimate of ‘actual’ use. Strengths of this study include the establishment of a longitudinal monitoring system and the systematic recording of multiple indicators of use, and detailed personal characteristics.

It may also be questioned if some girls did not use the cup at all, but allowed others, e.g. a sibling or friend to use it instead, creating the colour change; and we noted 2.6% of girls screened presented nurses with a cup that showed a colour change while their self-reports stated they did not use it. As the cup size was for girls, sharing with adult women would have been minimal due to potential leakage, but we acknowledge some sharing with siblings may have taken place. However, in focus group discussions participants feared cup use by another person may spread infection, and that they hid their cup [22]. During the nurse assessments, all girls denied sharing the cup with others.

## Conclusion

Our data provide objective physical evidence that schoolgirls, including young girls recently transitioning through menarche, are able and willing to embrace use of an insertable menstrual solution to manage their monthly periods in a rural African setting. Preliminary uptake was slow and peer support and mentoring was required; it may take up to 6 months before confidence to use is established. Comparison between self-reports and physical evidence suggests self-reported use likely over-inflates use. We found no evidence of high risk of damage or loss of product, and hygiene standards were reportedly maintained. Examination of safety when used for a longer duration, the barriers that result in a slow uptake and evaluations of the cost-benefit of menstrual cups over other products on health, wellbeing and schooling outcomes may help define menstrual cups’ potential role for managing girls’ and women’s menstrual needs in low and middle income countries. Menstrual cups have an upfront cost and may be scarce in impoverished areas. Some charities have developed cup distribution programs. However, efforts will be required to develop more sustainable marketing strategies to enable girls and women to access cups in such areas, with schemes for subsidies or pay-over-time in addition to peer-support development, especially for the first months, to enhance the uptake; to strengthen such activities and further research a cup coalition has been formed following a summit in January 2018 [48].

## Additional file

Additional file 1: **Figure S1.** Image of mooncup. **Figure S2.** Use of menstrual cup by self-report, colour change, or presence of clipped tail per study year quarter, Western Kenya, 2012-2013. **Figure S3.** Observed and reported menstrual cup hygiene among school girls, western Kenya. **Figure S4.** Menstrual cup use by verbal report at first report of cup colour change or at last nurse visit if no colour change, and as assessed by cup colour change by school, Western Kenya, 2012-2013. **Table S1.** Monitoring of menstrual cup use. **Table S2.** Examples of complaints and issues reported to the nurses on menstrual cup use by school girls, Western Kenya. **Table S3.** Factors associated with a verbal report of cup use during the study period among school girls in Western Kenya. **Table S4.** Factors associated with the presence of cup colour change during the study period among school girls in Western Kenya. **Table S5.** Factors associated with self-report of menstrual cup use among school girls with no cup colour change compared to self-report of cup use among school. (DOCX 7327 kb)
