# Supplementary material for: Use of menstrual cups among school girls: longitudinal observations nested in a randomised controlled feasibility study in rural western Kenya
Source: Reprod Health. 2018 Aug 17;15:139. doi: 10.1186/s12978-018-0582-8 (PMC6098596; doi:10.1186/s12978-018-0582-8)
Supplement: Supplementary file 1 — Figure S1. Image of mooncup. Figure S2. Use of menstrual cup by self-report, colour change, or presence of clipped tail per study year quarter, Western Kenya, 2012-2013. Figure S3. Observed and reported menstrual cup hygiene among school girls, western Kenya. Figure S4. Menstrual cup use by verbal report at first report of cup colour change or at last nurse visit if no colour change, and as assessed by cup colour change by school, Western Kenya, 2012-2013. Table S1. Monitoring of menstrual cup use. Table S2. Examples of complaints and issues reported to the nurses on menstrual cup use by school girls, Western Kenya. Table S3. Factors associated with a verbal report of cup use during the study period among school girls in Western Kenya. Table S4. Factors associated with the presence of cup colour change during the study period among school girls in Western Kenya. Table S5. Factors associated with self-report of menstrual cup use among school girls with no cup colour change compared to self-report of cup use among school. (DOCX 7327 kb) [file 12978_2018_582_MOESM1_ESM.docx]

**Additional file 1**

**Use of menstrual cups among school girls: longitudinal observations nested in a randomised controlled feasibility study in rural western Kenya**

**Figure S1.** Image of mooncup


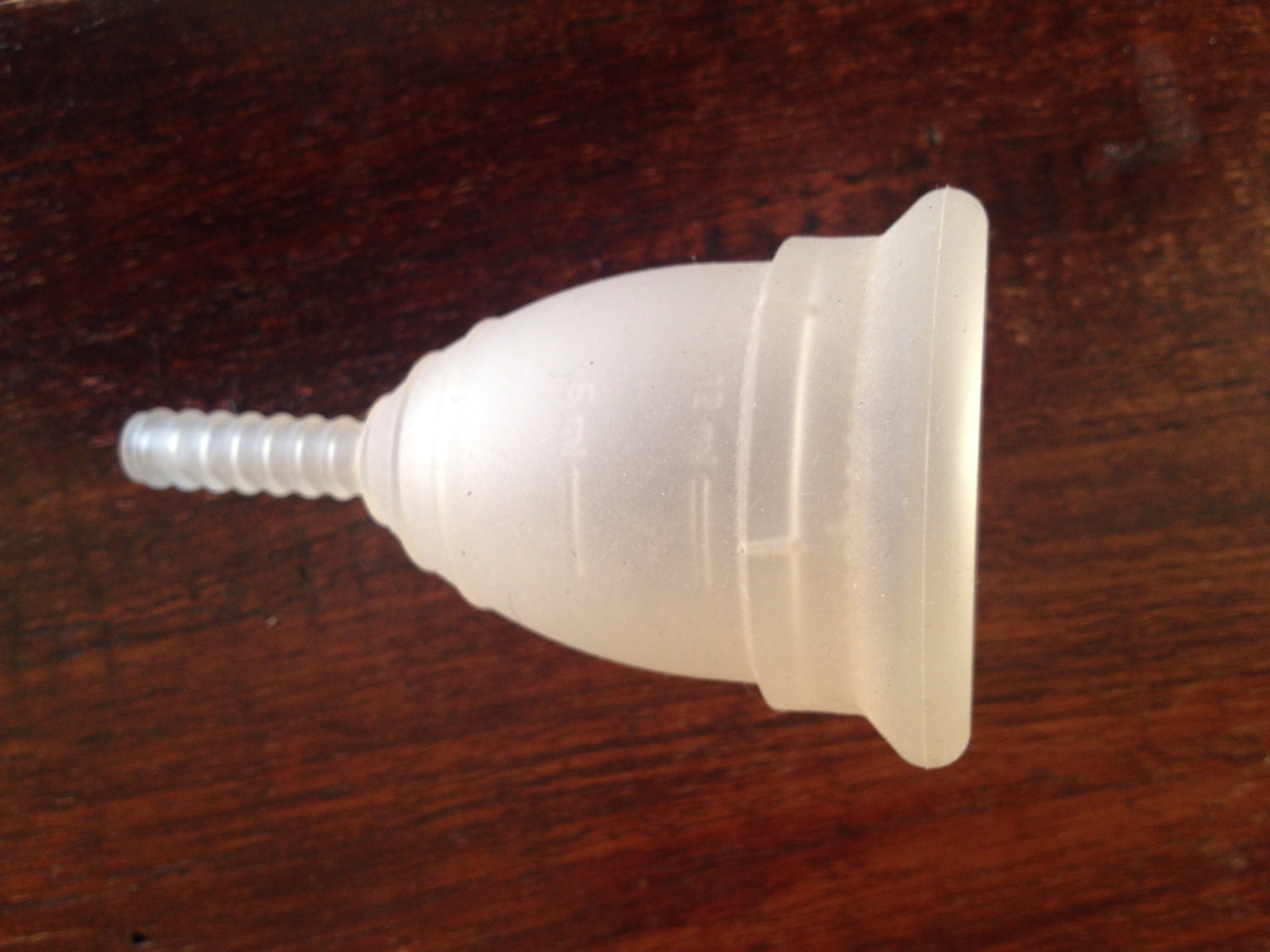


**Figure S2.** Use of menstrual cup by self-report, colour change, or presence of clipped tail per study year quarter, Western Kenya, 2012-2013

Note: If more than one nurse visit was made in the 3-month period, only information from the last visit was included

**Figure S3.** Observed and reported menstrual cup hygiene among school girls, western Kenya

*Observed by nurses during menstrual cup screening visits

†Before and after emptying of the menstrual cup

**Figure S4**. Menstrual cup use by verbal report at first report of cup colour change or at last nurse visit if no colour change, and as assessed by cup colour change by school, Western Kenya, 2012-2013

* Verbal response at the time of first report of cup colour change or at the last nurse assessment visit if the cup colour change did not occur; chi-square test comparing cup colour change among schools: p<0.001, chi-square test comparing verbal response among schools: p=0.184.

**Table S1.** Monitoring of menstrual cup use

During the monitoring visits to the nurse, girls in the menstrual cup arm were asked specific questions on their cup use, if they dropped it, shared use with another person, and how they cleaned it. Girls were required to bring in their cup (barring current use) for physical examination at each visit, summarised below.

| Look | Describe | Ask |
| --- | --- | --- |
| What colour is the menstrual cup? | Clear/white, pink, yellow, green | Anything else |
| General wear and tear | None, small from use, damaged | Why damaged |
| Is the rim structure damaged? | Complete, small split, large split | Why split |
| Is the cup structure damaged? | Complete, cracked, hole/s | Why damaged |
| Is the tail length corrected? | Long (untrimmed), medium, short, none | Trimming issues |
| Is the tail structure damaged? | End smooth, end ragged/split | Why damaged |
| Is there detritus (faeces, paper)? | No deposits, grainy deposits, thick deposits | Describe |
| Smell | None, blood, other | Describe other |

**Table S2.** Examples of complaints and issues reported to the nurses on menstrual cup use by school girls, Western Kenya

| Complaint or issue |
| --- |
| It is challenging inserting and removing the cup during the initial states |
| Painful during insertion |
| Some girls found it disgusting especially when removing and emptying the cup when the water is not available or limited. |
| Some parents/guardian did not allow their girls to use mooncup (e.g. some had the misconception that use of the menstrual cup will make girls urge for sex |
| Some girls felt embarrassed to boil the cup and it was challenging to find an item (e.g. tin) for boiling the cup. |
| Some girls felt uncomfortable when using mooncup |
| Some girls complained of unhygienic latrines and lack of proper structures in school latrines; (unlockable latrine doors hence girls forced to hold onto doors while in the latrines.) |
| Where do we empty the menstrual cup for homes without latrines |
| Spillage of blood during removal (Spillage of blood during removal was asked by our participants and the experienced peer-group girls demonstrated the angle at which it should be removed to avoid spillage). |
| What do I use in case it falls on the latrine floor as am changing? |
| Mine is leaking even when it is not full |
| What if there is no water in the school how do I clean my hands after changing |

**Table S3.** Factors associated with a verbal report of cup use during the study period among school girls in Western Kenya

| Characteristic | **No of visits (N=849)** | **verbal report of cupuse (% of category)** | **p-value (χ^2^-test)** | **Risk ratio, 95% CI***  **Univariate analysis** | **p-value** |
| --- | --- | --- | --- | --- | --- |
| Class at time of enrolment | | |  |  |  |
| 5 or 6 | 282 | 255 (90.4) | 0.276 | 1.00, 0.97-1.04 | 0.846 |
| 7 or 8 | 567 | 525 (92.6) |  | Reference |  |
| Age at enrolment (years) | |  |  |  |  |
| 14 | 438 | 407 (92.9) | 0.168 | Reference |  |
| 15 | 328 | 301 (91.8) |  | 0.69, 0.39-1.24 | 0.216 |
| 16 | 83 | 72 (86.8) |  | 0.54, 0.21-1.37 | 0.192 |
| Socioeconomic status household of girls | |  |  |  |  |
| Poorest 2 quintiles | 79 | 74 (93.7) | 0.640 | Reference |  |
| Higher 3 quintiles | 627 | 577 (92.0) |  | 1.17, 0.45-3.08 | 0.744 |
| No information | 143 | 129 (90.2) |  | 1.34, 0.44-4.08 | 0.610 |
| Material used for menstruation before enrolment | | | |  |  |
| Used some pads | 96 | 85 (88.5) | 0.205 | 0.80, 0.44-1.44 | 0.449 |
| Cloth/other | 753 | 695 (92.3) |  | Reference |  |
| Time since menarche (enrolment) | | |  |  |  |
| < 1 year | 232 | 224 (96.6) | 0.002 | 2.03, 1.04-3.94 | **0.037** |
| ≥ 1 year | 617 | 556 (90.1) |  | Reference |  |
| Enrolment year |  |  |  |  |  |
| 2012 | 611 | 570 (93.3) | 0.015 | Reference | 0.060 |
| 2013 | 238 | 210 (88.2) |  | 0.96, 0.92-1.00 |  |
| Enrolled before/after peer education | | |  |  |  |
| Before | 494 | 456 (92.3) | 0.584 | Reference | 0.263 |
| After | 355 | 324 (91.3) |  | 0.70, 0.38-1.30 |  |
| Follow up time at time of visit |  |  |  |  |  |
| <3 months | 208 | 174 (83.7) | <0.001 | 0.38, 0.19-0.79 | **0.010** |
| 3-<6 months | 229 | 217 (94.8) |  | 1.44, 0.59-3.49 | 0.421 |
| 6-<9 months | 176 | 171 (97.2) |  | 1.80, 0.65-4.98 | 0.255 |
| 9+ months | 236 | 218 (92.4) |  | Reference |  |
| School number |  |  |  |  |  |
| 1 | 43 | 30 (69.8) | <0.001 | 0.25, 0.09-0.74 | **0.012** |
| 2 | 69 | 68 (98.6) |  | 7.30, 0.79-67.46 | 0.080 |
| 3 | 51 | 46 (90.2) |  | Reference |  |
| 4 | 133 | 122 (91.7) |  | 1.21, 0.40-3.64 | 0.737 |
| 5 | 93 | 90 (96.8) |  | 3.24, 0.75-13.93 | 0.114 |
| 6 | 208 | 203 (97.6) |  | 4.44, 1.15-17.10 | **0.030** |
| 7 | 79 | 79 (100.0) |  |  |  |
| 8 | 63 | 43 (68.3) |  | 0.23, 0.08-0.69 | **0.008** |
| 9 | 79 | 72 (91.1) |  | 1.12, 0.37-3.34 | 0.841 |
| 10 | 31 | 27 (87.1) |  | 0.74, 0.25-2.15 | 0.576 |

* Generalized estimating equations with a logit link and exchangeable correlation. All models were adjusted for school. Factors examined but not significant: Socioeconomic status of the school catchment area, age at menarche, days of bleeding, amount of bleeding and cramps when menstruating. Multivariate models could not be conducted because models would not converge.

**Table S4.** Factors associated with the presence of cup colour change during the study period among school girls in Western Kenya

| Characteristic | **Prevalence**  **N=192**  **(% of sample size)** | **Uptake of cup (% of category)** | **p-value (χ^2^-test)** | **Risk ratio, 95% CI***  **Univariate analysis** | **p-value** | **Adjusted Risk ratio, 95% CI***  **Multivariate analysis** | **p-value** |
| --- | --- | --- | --- | --- | --- | --- | --- |
| Class at time of enrolment | | |  |  |  |  |  |
| 5 or 6 | 61 (31.8) | 42 (68.9) | 0.680 | 0.96, 0.78-1.18 | 0.698 |  |  |
| 7 or 8 | 131 (68.2) | 91 (71.8) |  | Reference |  |  |  |
| Age at enrolment (years) | |  |  |  |  |  |  |
| 14 | 93 (48.4) | 68 (73.1) | 0.437 | Reference |  |  |  |
| 15 | 80 (41.7) | 53 (66.3) |  | 0.91, 0.76-1.07 | 0.255 |  |  |
| 16 | 19 (9.9) | 15 (79.0) |  | 1.08, 0.70-1.65 | 0.725 |  |  |
| Socioeconomic status household of girls | |  |  |  |  |  |  |
| Poorest 2 quintiles | 22 (11.5) | 14 (63.6) | 0.672 | 0.93, 0.58-1.48 | 0.745 |  |  |
| Higher 3 quintiles | 138 (71.9) | 100 (72.5) |  | 1.05, 0.83-1.34 | 0.669 |  |  |
| No information | 32 (16.7) | 22 (68.8) |  | Reference |  |  |  |
| Material used for menstruation before enrolment | | | |  |  |  |  |
| Used some pads | 169 (88.0) | 120 (71.0) | 0.887 | Reference |  |  |  |
| Cloth/other | 23 (12.0) | 16 (69.6) |  | 0.98, 0.77-1.24 | 0.866 |  |  |
| Time since menarche (enrolment) | | |  |  |  |  |  |
| < 1 year | 51 (26.6) | 43 (84.3) | 0.013 | 1.28, 1.05-1.56 | **0.016** | 1.29, 1.04-1.60 | **0.023** |
| ≥ 1 year | 141 (73.4) | 93 (66.0) |  | Reference |  |  |  |
| Enrolment year |  |  |  |  |  |  |  |
| 2012 | 126 (65.6) | 89 (70.6) | 0.933 | Reference |  |  |  |
| 2013 | 66 (34.4) | 47 (71.2) |  | 1.01, 0.82-1.24 | 0.938 |  |  |
| Enrolled before/after peer education | | |  |  |  |  |  |
| Before | 102 (53.1) | 70 (68.6) | 0.474 | Reference | 0.596 |  |  |
| After | 90 (46.9) | 73 (73.3) |  | 1.07, 0.84-1.37 |  |  |  |
| Total duration of participation in study | | |  |  |  |  |  |
| <3 months | 23 (12.0) | 8 (34.8) | <0.001 | 0.43, 0.27-0.68 | **<0.001** | 0.63, 0.43-0.93 | **0.019** |
| 3-<6 months | 53 (27.6) | 34 (64.2) |  | 0.80, 0.68-0.94 | **0.007** | 1.10, 0.91-1.33 | 0.348 |
| 6-<9 months | 35 (18.2) | 29 (82.9) |  | 1.03, 0.89-1.20 | 0.674 | 1.20, 1.01-1.42 | **0.041** |
| 9+ months | 81 (42.2) | 65 (80.3) |  | Reference |  |  |  |
| Number of visits made in study | |  |  |  |  |  |  |
| 1-3 visits | 74 (38.5) | 37 (50.0) | <0.001 | 0.60, 0.44-0.81 | **0.001** | 0.63, 0.45-0.88 | **0.007** |
| 4+ visits | 118 (61.5) | 99 (83.9) |  | Reference |  | Reference |  |

*Generalized linear regression with a log link and binomial distribution. All models were adjusted for school as a cluster. Factors examined but not significant: Socioeconomic status of the school catchment area, age at menarche, days of bleeding, amount of bleeding and cramps when menstruating.

**Table S5.** Factors associated with self-report of menstrual cup use among school girls with no cup colour change compared to self-report of cup use among school girls with cup-colour change, Western Kenya

| Characteristic | **Prevalence**  **N=181**  **(% of sample size)** | **Self report of cup use**  **and no cup colour change at last visit**  **N=50 (% in category)** | **Self report of cup use at time of colour change**  **N=131 (% in category)** | **p-value (χ^2^-test)** |
| --- | --- | --- | --- | --- |
| Class at time of enrolment | | |  |  |
| 5 or 6 | 57 (6.6) | 18 (31.6) | 39 (68.4) | 0.420 |
| 7 or 8 | 124 (24.9) | 32 (25.8) | 92 (74.2) |  |
| Age at enrolment (years) | |  |  |  |
| 14 | 91 (50.3) | 24 (26.4) | 67 (73.6) | 0.564 |
| 15 | 74 (40.9) | 23 (31.1) | 51 (68.9) |  |
| 16 | 16 (8.8) | 3 (18.8) | 13 (81.3) |  |
| Socioeconomic status household of girls | |  |  |  |
| Poorest 2 quintiles | 20 (11.1) | 6 (30.0) | 14 (70.0) | 0.907 |
| Higher 3 quintiles | 131 (72.4) | 35 (26.7) | 96 (73.3) |  |
| No information | 30 (16.6) | 9 (30.0) | 21 (70.0) |  |
| Time since menarche (enrolment) |  |  |  |  |
| < 1 year | 51 (28.2) | 8 (15.7) | 43 (84.3) | 0.024* |
| ≥ 1 year | 130 (71.8) | 42 (32.3) | 88 (67.7) |  |

* Generalized linear regression with a log link and binomial distribution with school as cluster: risk ratio 0.49, 0.29-0.82, p=0.007; girls within their first year of the start of menstruation were less likely to report cup use without colour change compared to girls who started menstruation more than a year ago.
